# Privy Vaults

**Published:** 1875-09

**Authors:** 


### PRIVY VAULTS.

Cesspools and privy vaults are great nuisances and ought not to exist. They would not exist, but for the inordinate lazyness of mankind. It is easier for a lazy man to dig a deep pit and then go on from year to year, filling it with excrement, making it a horrible, pestilential spot in the fair universe, than to leave the pit undug and sprinkle a little dry earth over each days' accumulations. But when the cesspool exists, its contents may be rendered comparatively inodorous by frequent additions of plaster, or ground, unburned gypsum, or diluted oil of vitrol, or solution of copperas. Probably the easiest way of using the contents for manure is to throw fine earth into the cesspool until a thick mass is formed, and to scoop this out and cover it with a quantity of dry soil gathered in readiness for it. This easiest absorbs all the liquid and all the gaseous matter, and if kept covered from rain for a few days, it will be in excellent condition to spread over meadows or fallows to be prepared for Fall crops.

